# Role of Lymphocyte Activation Gene-3 (Lag-3) in Conventional and Regulatory T Cell Function in Allogeneic Transplantation

**DOI:** 10.1371/journal.pone.0086551

**Published:** 2014-01-27

**Authors:** Emanuela I. Sega, Dennis B. Leveson-Gower, Mareike Florek, Dominik Schneidawind, Richard H. Luong, Robert S. Negrin

**Affiliations:** 1 Department of Medicine, Stanford University, Stanford, California, United States of America; 2 Department of Comparative Medicine, Stanford University, Stanford, California, United States of America; Beth Israel Deaconess Medical Center, Harvard Medical School, United States of America

## Abstract

Lag-3 has emerged as an important molecule in T cell biology. We investigated the role of Lag-3 in conventional T cell (Tcon) and regulatory T cell (Treg) function in murine GVHD with the hypothesis that Lag-3 engagement diminishes alloreactive T cell responses after bone marrow transplantation. We demonstrate that Lag-3 deficient Tcon (Lag-3^−/−^ Tcon) induce significantly more severe GVHD than wild type (WT) Tcon and that the absence of Lag-3 on CD4 but not CD8 T cells is responsible for exacerbating GVHD. Lag-3^−/−^ Tcon exhibited increased activation and proliferation as indicated by CFSE and bioluminescence imaging analyses and higher levels of activation markers such as CD69, CD107a, granzyme B, and Ki-67 as well as production of IL-10 and IFN-g early after transplantation. Lag-3^−/−^ Tcon were less responsive to suppression by WT Treg as compared to WT Tcon. The absence of Lag-3, however, did not impair Treg function as both Lag-3^−/−^ and WT Treg equally suppress the proliferation of Tcon *in vitro* and *in vivo* and protect against GVHD. Further, we demonstrate that allogeneic Treg acquire recipient MHC class II molecules through a process termed trogocytosis. As MHC class II is a ligand for Lag-3, we propose a novel suppression mechanism employed by Treg involving the acquisition of host MHC-II followed by the engagement of Lag-3 on T cells. These studies demonstrate for the first time the biologic function of Lag-3 expression on conventional and regulatory T cells in GVHD and identify Lag-3 as an important regulatory molecule involved in alloreactive T cell proliferation and activation after bone marrow transplantation.

## Introduction

Allogeneic hematopoietic cell transplantation (HCT) is an effective treatment for patients with a broad range of hematological malignancies, but is limited by graft-versus-host-disease (GVHD). Acute GVHD is caused by alloreactive donor-derived T cells reacting to host antigens expressed by antigen presenting cells (APC’s) leading to activation and proliferation of T cells resulting in tissue damage, primarily in the skin, gastrointestinal tract, and liver [1], [2]. Since donor T cells are the main effector cell population mediating GVHD, controlling their alloreactivity while maintaining graft-versus-tumor (GVT) effects would improve outcomes and allow for a wider use of HCT.

Different regulatory cell populations such as (CD4^+^CD25^+^FoxP3^+^) regulatory T cells (Treg), natural killer T (NKT) cells, anti-inflammatory cytokines (i.e. IL-10, TGF-β), and inhibitory molecules (i.e. CTLA-4 and PD-1) involved in controlling the proliferation and activation of alloreactive T cells have been identified and found to play important roles in GVHD pathophysiology [3], [4], [5], [6], [7], [8], [9], [10], [11], [12].

Recently, lymphocyte-activation gene 3 (Lag-3) has emerged as another important molecule that regulates T cell function. Lag-3 is a transmembrane protein, highly homologous to CD4 structurally, but with less than 20% identity at the amino acid level [13], [14]. LAG-3 is not only expressed on different subsets of T cells (CD4, CD8, γδ T cells, Treg) but also on B cells, NK cells and plasmacytoid DC [15], [16], [17], [18], [19]. The known ligand for Lag-3 is MHC class II, to which it binds with higher affinity than CD4 [20]. Similar to CTLA-4 and PD-1, Lag-3 negatively regulates cellular proliferation, activation, and homeostasis of T cells, and has been reported to play a role in Treg suppressive function [14], [19], [21]. Lag-3 is involved in maintaining the tolerogenic state of CD8 T cells in models of self and tumor tolerance and synergizes with PD-1 in maintaining CD8 exhaustion during chronic viral infection [22], [23]. Together with PD-1 and TGF-β, Lag-3 contributes to CD8 T cell tolerance induced by allogeneic BMT with anti-CD40L antibody [24].

Given that Lag-3 is a negative regulator of proliferation and activation of T cells, we hypothesized that Lag-3 engagement on donor T cells may affect allogeneic T cell activation and proliferation impacting GVHD pathophysiology.

Our data demonstrate that T cells lacking Lag-3 have enhanced donor T cell alloreactivity with increased proliferation and enhanced ability to induce GVHD. Furthermore, we demonstrate that Lag-3^−/−^ T cells are less responsive to suppression by WT Treg and that Lag-3^−/−^ Treg are as potent as WT Treg in suppressing donor T cell proliferation. Lastly, we propose that Treg function in part through acquisition of recipient MHC class II molecules and interact through Lag-3 expressed on donor T cells.

## Materials and Methods

### Ethics Statement

All animal studies were approved by the Institutional Animal Care and Use Committee of Stanford University (protocol #10269).

### Animals

C57BL/6 (H-2b) and Balb/c (H-2d) mice were purchased from Jackson Laboratory. Lag-3^−/−^ mice were a gift from Yueh-Hsiu Chien (Stanford University). Luciferase expressing (*luc^+^*) C57BL/6 mice (H-2b, CD45.1^+^, Thy1.1^+^) were created as previously described [6]. *Luc^+^*Lag-3^−/−^ mice were generated by breeding Lag-3^−/−^ mice and *luc^+^* C57BL/6 mice for three generations.

### Cell Isolation and Sorting

Single cell suspensions from spleen and lymph nodes (LN) were enriched first for CD4^+^ and then CD8^+^ T cells with anti-CD4 and anti-CD8 magnetic beads, respectively, using the MidiMACS system (Miltenyi Biotech). For conventional T cells (Tcon), CD4 and CD8 T cells were mixed so that the CD4:CD8 ratio was 2∶1. T cell depleted bone marrow (TCD-BM) was prepared by flushing bones and depleting T cells with anti-CD4 and anti-CD8 magnetic beads. To isolate regulatory T cells (Treg), single cell suspensions from spleen and LNs were enriched for CD25^+^Tcells and sorted for CD4^+^CD25^hi^ cells on a FACS Aria flow cytometer (Becton Dickinson) to a purity of 96–98%.

### Flow Cytometric Analyses

The following antibodies were purchased from BD Pharmingen, eBiosciences or BioLegend: CD4 (GK1.5), CD8 (53–6.7), CD45.1 (A20), Thy1.1 (H1S51), CD25 (PC61), H-2Kb (AF6-88.5), H-2Kd (34-2-12), Lag-3 (C9B7W), I-A^d^ (39-10-8), I-A^b^ (KH74), Foxp3 (FJK-16s), CD62L (MEL-14), CD69 (H1.2F3), CD107a (1D4B), Ki-67 (SolA15), IFN-γ (XMG1.2), IL-10 (JES5-16E3). Foxp3 staining was performed with the anti-mouse/rat Foxp3 staining set (eBiosciences). Dead cells were stained with Live/Dead Fixable Aqua Dead Cell Staining kit (Invitrogen). For intracellular cytokine staining, 250 µg brefeldin A (Biolegend) was injected i.p. into mice 6 hours prior to harvesting spleen and LNs for analysis. To maintain the retention of intracellular cytokines, brefeldin A was included in all media and staining buffers until cells were fixed and permeabilized. No additional *ex vivo* T cell stimulation was done. Analysis was performed on a 4-laser LSRII flow cytometer (Becton Dickinson).

### GVHD Model

Balb/c recipients were lethally irradiated with 8 Gy split into 2 doses, 4 hours apart. To induce GVHD, 5×10^6^ TCD-BM (C57BL/6) together with 0.5−1×10^6^ Tcon were injected into recipient mice via tail vein on day 0. In the experiments involving Treg, 5×10^6^ TCD-BM were co-injected with 5×10^5^ Treg cells on day 0 followed by 1×10^6^ Tcon on day 2. Transplanted animals were kept in autoclaved cages with antibiotic water (sulfamethoxazole-trimethropim, Shein Pharmaceutical). Clinical GVHD scoring was based on activity, posture, weight loss, skin lesions, and fur changes (ruffled versus normal).

### *In vivo* and *ex vivo* Bioluminescence Imaging

*In vivo* bioluminescence imaging (BLI) was performed as previously described [3]. Briefly, mice were injected intraperitoneally with luciferin (150 µg/g body weight) and imaged ten minutes later with an IVIS Spectrum charge-coupled device (CCD) imaging system (Xenogen). Images were analyzed with Living Image software (Xenogen) and Igor Pro Carbon (Wavemetrics).

### *In vivo* CFSE Proliferation Assay

CFSE proliferation analysis was performed using CellTrace™ CFSE Cell Proliferation Kit (Invitrogen). Tcon were labeled with 5 µM CellTrace™ CFSE in PBS for 5 min at 37°C, quenched by addition of cold RPMI (Invitrogen) with 10% FCS, and washed 4 times to remove excess CFSE. CFSE-labeled Tcon together with 5×10^6^ TCD-BM were injected into lethally irradiated Balb/c mice on day 0. On day 3, CFSE staining of donor T cells isolated from spleen and lymph nodes was analyzed by FACS.

### Mixed Leukocyte Reaction (MLR)

Irradiated (30 Gy) Balb/c splenocytes (stimulators) were plated together with allogeneic Tcon (responders) at a 2∶1 ratio and different doses of Treg isolated from either WT or Lag-3^−/−^ mice. Following incubation for 96 hours, cells were pulsed with 1 µCi/well of [3H]-thymidine and thymidine incorporation was measured with a Wallac Betaplate counter (Perkin-Elmer).

### Statistical Analysis

Statistical analyses were performed with GraphPad Prism software. The log-rank test was used to compare differences in animal survival. Two way ANOVA was used to compare curves for the whole body BLI experiments. All other comparisons were performed with the 2-tailed Student t test and P≤0.05 was considered statistically significant.

## Results

### Lag-3 is Up-regulated on Activated T Cells

**Figure 1:**
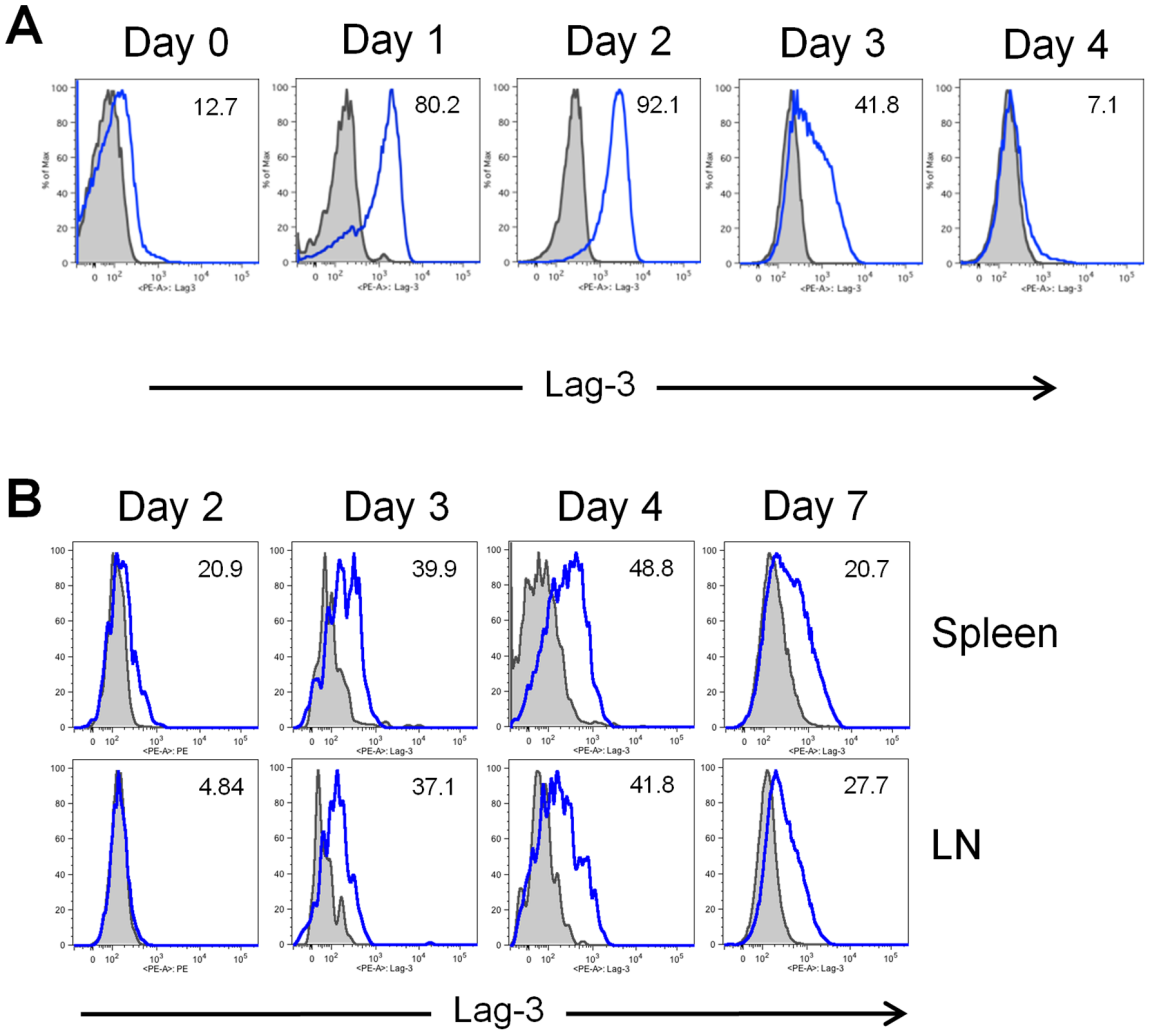
Surface expression of Lag-3 increases upon T cell activation *in vitro* and *in vivo*. (A) T cells were incubated with anti-CD3/anti-CD28 activation beads in the presence of 1000 U/mL hrIL-2. Lag-3 expression was assessed over a period of 7 days. The grey solid histograms represent cells stained with isotype control Ab. Cells were gated on CD4^+^ and the numbers in the right side corner represent the percentage of Lag-3 positive cells. Data is representative of 3 independent experiments. (B) Balb/c (H-2K^d^) recipients were co-transplanted with 5×10^6^ TCD-BM and 1×10^6^ Tcon from C57Bl/6 (H-2K^b^) mice. At indicated time points after transplantation, donor T cells were re-isolated from spleen and LN and analyzed for Lag-3 expression. Grey histograms represent cells stained with isotype controls. Cells were gated on donor CD4^+^ cells and the numbers in the right side corner represent the percentage of Lag-3 positive cells. Data is representative of two independent experiments.

Previous work from our laboratory has demonstrated that the first three days after transplantation are critical for the initiation of GVHD by donor-derived alloreactive T cells [25]. During the first several days following transplantation, T cells infiltrate secondary lymphoid organs, interact with host APCs, become activated, proliferate, and differentiate into effector T cells. Lag-3 expression is up-regulated on the surface of T cells immediately after their activation *in vitro* (Fig. 1A). Therefore, we examined whether similar up-regulation of Lag-3occurs *in vivo* after transplantation. To determine the expression pattern of Lag-3 on donor T cells, lethally irradiated Balb/c (H-2K^d^) recipients were transplanted with 1×10^6^ conventional CD4^+^ and CD8^+^ T cells (Tcon) together with 5×10^6^ TCD-BM from C57BL/6 (H-2K^b^) donors. Lymph nodes (LN) and spleen were harvested at various time points for analysis. Lag-3 expression was detected by day 2 on donor derived T cells infiltrating the spleen and day 3 in LNs, and increased over the next two days with maximum expression observed on day 4, with approximately 50% of splenic and 40% of nodal donor T cells expressing Lag-3. By day 7, in agreement with the *in vitro* data, fewer T cells expressed Lag-3 on their surface.

### Lag-3^−/−^ Tcon Accelerates GVHD

**Figure 2:**
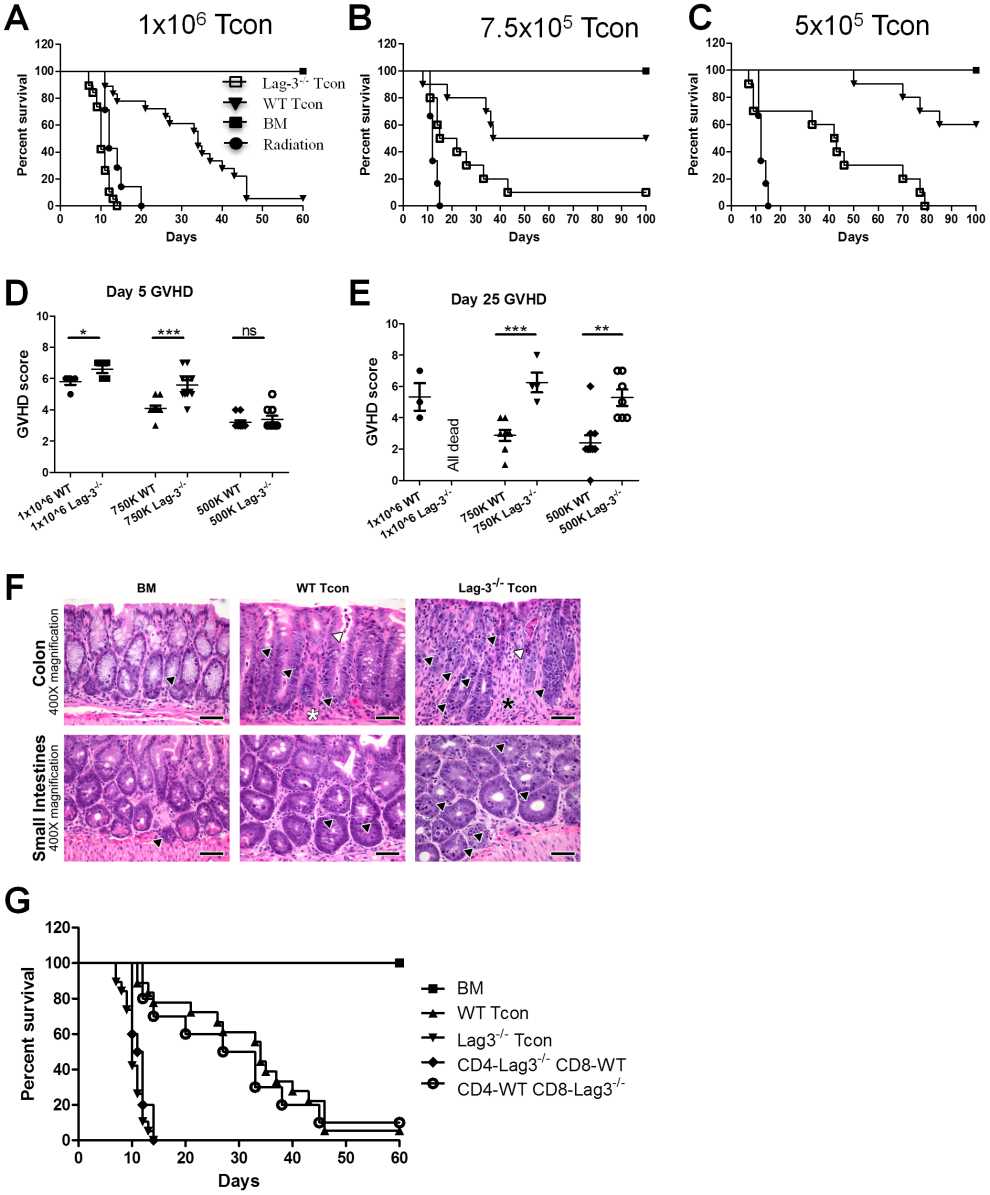
Lag-3^−/−^ Tcon accelerates GVHD. Lethally irradiated Balb/c recipients were transplanted with 5×10^6^ TCD-BM and various doses of Tcon from either Lag-3^−/−^ or WT C57Bl/6 mice and monitored for survival (A–C) and GVHD score (D–E). (A) Mice were infused with TCD-BM and 1×10^6^ Tcon (n = 15/group, P = 0.009), (B) TCD-BM and 7.5×10^5^ Tcon (n = 10/group, P<0.001), and (C) TCD-BM and 5×10^5^ Tcon (n = 10/group, P<0.001). P value is calculated for survival rate differences between mice receiving Lag-3^−/−^ Tcon and WT Tcon. Data in graphs are pooled from two independent experiments. (D) and (E) represent the GVHD score of mice 5 days (D) and 25 days (E) after transplantation. Mice receiving TCD-BM and Lag-3^−/−^ Tcon had a significantly higher GVHD score than mice receiving TCD-BM and WT Tcon (P<0.005). (F) Histopathologic analysis of GVHD target organs isolated at day 8 after transplant. High magnification photomicrographs of colon (upper panels) and small intestine (lower panels) reveal a more severe GVHD in mice transplanted with Lag-3^−/−^ Tcon as evidenced by increased inflammatory cell infiltrate in the lamina propria (black asterisk) and intestinal gland lumina (white arrowhead), loss of microarchitecture of intestinal glands that are lined by poorly-differentiated epithelial cells and a larger number of apoptotic bodies within the epithelial layer (black arrowhead). H&E stain, scale bar = 50 microns. (G) Mice received 5×10^6^ TCD-BM and 1×10^6^ different combinations of CD4 and CD8 T cells. One group of mice received Lag-3^−/−^ CD4+ WT-CD8 T cells (n = 10), one received WT-CD4+ Lag-3^−/−^ CD8 T cells (n = 10, P<0.0001), one group received Lag-3^−/−^CD4+Lag-3^−/−^CD8T cells (Lag-3^−/−^ Tcon, n = 15) and the last group received WT-CD4+WT-CD8 T cells (WT Tcon, n = 15). P values are calculated for survival rate differences between mice receiving Lag-3^−/−^ Tcon and WT-CD4+Lag-3^−/−^CD8 T cells. Data are pooled from two independent experiments.

The timing of Lag-3 up-regulation on the surface of Tcon coincides with the Tcon proliferation phase during acute GVHD development; therefore, we suspected that Lag-3 might play an important role in GVHD initiation. To test this hypothesis, we used donor T cells from either Lag-3^−/−^ or WT mice and assessed their ability to induce aGVHD. Lethally irradiated Balb/c recipients were injected with 5×10^6^ TCD-BM from C57BL/6 mice together with 1×10^6^, 7.5×10^5^ or 5×10^5^ Tcon (Fig. 2). Mice were monitored for survival and GVHD metrics (fur, weight loss, activity, and posture). Mice receiving Lag-3^−/−^Tcon had significantly decreased survival at each T cell dose, with a median survival time of 34 days for 1×10^6^ WT Tcon compared to a median survival of 9 days for 1×10^6^ Lag-3^−/−^ Tcon (P = 0.009; Fig. 2A). The difference in survival was even more apparent when less severe GVHD was induced by transferring lower numbers of Tcon. Mice receiving 7.5×10^5^ Lag-3^−/−^ Tcon had a median survival of only 18.5 days, compared to a median survival of 68.5 days in mice receiving 7.5×10^5^ WT Tcon (P<0.0001) (Fig. 2B). Similarly, all mice receiving 5×10^5^ Lag-3^−/−^ Tcon died from GVHD, with a median survival of 42.5 days, while 60% of mice receiving 5×10^5^ WT Tcon survived past 100 days (P = 0.0002) (Fig. 2C). GVHD scores for Lag-3^−/−^ Tcon recipients were significantly higher than for those receiving WT Tcon at early and late time points (Day 5, Fig. 2D; day 25, Fig. 2E). In addition to the clinical parameters that indicate higher GVHD scores for Lag-3^−/−^ Tcon recipients, histopathological analysis of large and small intestine at day 8 after transplant also reveal a more severe GVHD in the absence of Lag-3 on Tcon. As shown in Fig. 2F, the colon of Lag-3^−/−^ Tcon recipients lost the normal microarchitecture of the large intestinal glands, the goblet cells that normally line the intestinal glands were replaced by poorly-differentiated epithelial cells with evidence of hyperplasia, there was a greater inflammatory cell infiltrate in both the lamina propria and the intestinal gland lumina, and a larger number of apoptotic bodies within the epithelial layer. There was a more severe loss of the crypt microarchitecture of the small intestine when mice were transplanted with Lag-3^−/−^ Tcon. The Paneth cells lining the crypt were replaced with hyperplastic, poorly differentiated epithelial cells with marked basophilia and mitoses, and, similar to the colon, a larger number of apoptotic bodies within the crypt epithelium were present. These histopathological findings together with the clinical score and survival data indicate that GVHD is significantly more severe in the absence of Lag-3 on Tcon indicating that Lag-3 plays an important role in limiting alloreactive T cell responses after BMT.

Since Tcon are a mixture of CD4 and CD8 T cells and since both populations are known to express Lag-3 on their surface, we assessed the importance of Lag-3 expression on CD4 and CD8 T cells separately. Mice were co-transplanted with TCD-BM and 1×10^6^ Tcon on day 0. Groups of mice received Lag-3^−/−^CD4 with WT-CD8, WT-CD4 with Lag3^−/−^CD8 cells, or Lag-3^−/−^ CD4 and CD8 cells, or WT CD4 and CD8 T cells. As indicated in Fig. 2G, mice receiving Lag-3^−/−^CD4 with WT-CD8 T cells showed similar survival and GVHD symptoms as mice receiving both Lag-3^−/−^ CD4 and CD8 T cells suggesting that the absence of Lag-3 on CD4 T cells is responsible for the observed increased GVHD pathogenesis. Conversely, mice receiving WT-CD4 with Lag-3^−/−^CD8 T cells had less severe GVHD and showed significant increase in median survival (P = 0.02) when compared with mice receiving Lag-3^−/−^Tcon, suggesting that Lag-3 engagement on CD8 T cells has a smaller influence on limiting the alloreactive T cell response after BMT.

### Regulatory T Cells are Less Able to Control Lag-3^−/−^ Tcon

**Figure 3:**
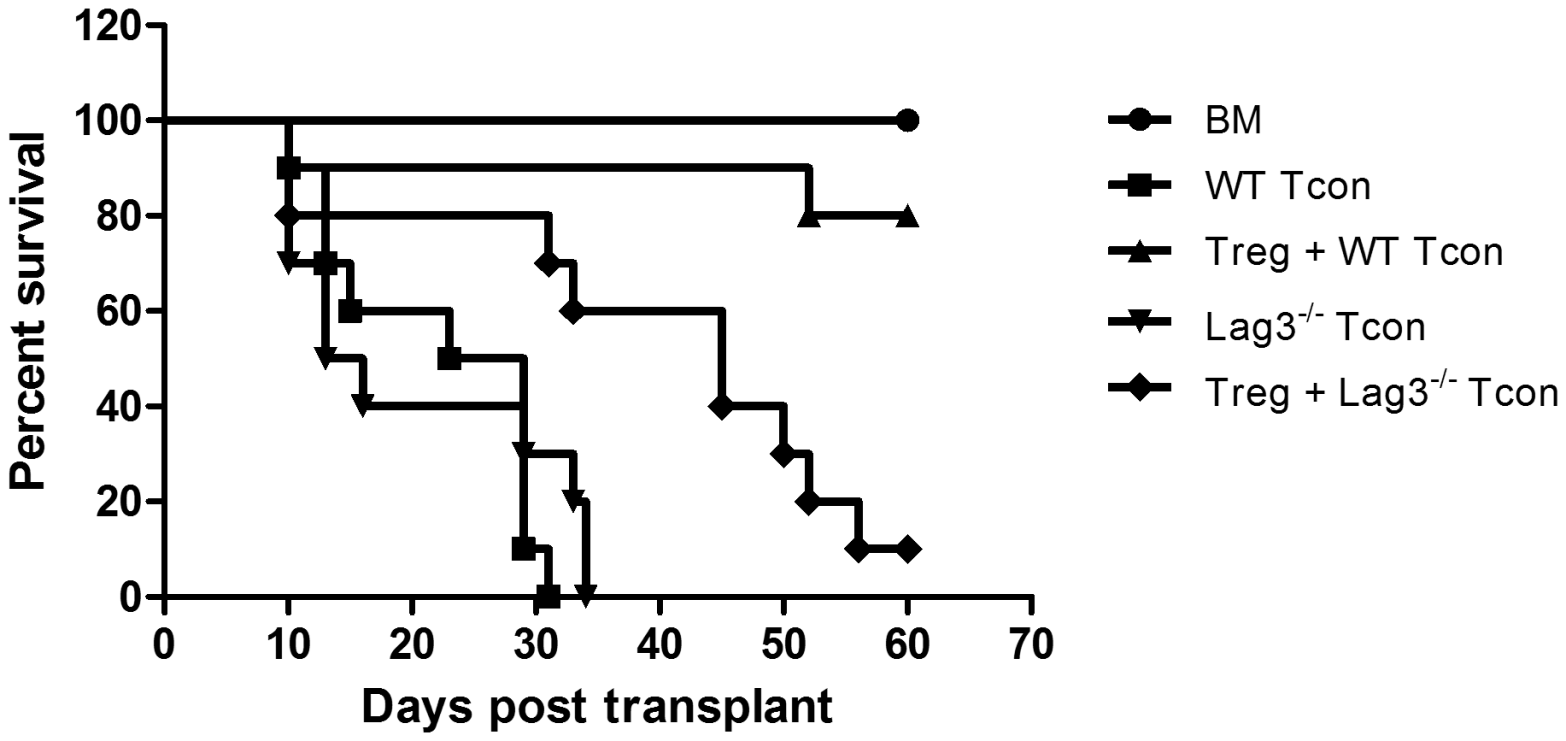
Regulatory T cells are less able to control Lag-3^−/−^ Tcon. Lethally irradiated Balb/c recipients were transplanted with 5×10^6^ TCD-BM and 5×10^5^ WT Treg on day 0 followed by infusion of 1×10^6^ Lag-3^−/−^ or WT Tcon on day 2. WT Treg provided less protection against GVHD induced by Lag-3^−/−^ Tcon as compared to WT Tcon. (n = 10, Data in graph are pooled from two independent transplants; P<0.001 for Treg+WT Tcon vs. WT Tcon, P = 0.03 for Treg+Lag-3^−/−^ Tcon vs. Lag-3^−/−^ Tcon and P = 0.003 for Treg+WT Tcon vs. Treg+Lag-3^−/−^ Tcon).

Treg are known to reduce GVHD severity and mortality by suppressing Tcon proliferation [4]. To investigate whether the addition of WT Treg would rescue mice from the severe GVHD caused by Lag-3^−/−^Tcon, 5×10^5^ highly purified CD4^+^CD25^+^Foxp3^+^ Treg from C57BL/6 mice were co-transferred on day 0 with 5×10^6^ TCD-BM, followed by 1×10^6^ Lag-3^−/−^ or WT Tcon on day 2. We chose this schedule based on previously published results from our laboratory which showed that a dose of 1×10^6^ Tcon induces lethal GVHD whether Tcon were transferred at day 0 or day 2 post BMT and that Treg infused 2 days before Tcon were able to suppress Tcon proliferation and protect from GVHD even at 10∶1 ratio of Tcon:Treg [4]. Therefore, this schedule allowed us to use a lower number of Tregs while still providing protection against GVHD. As indicated in Figure 3, administering 1×10^6^ WT or Lag-3^−/−^ Tcon two days after BMT induces acute lethal GVHD. However, the difference in GVHD severity induced by the two Tcon populations was smaller than the difference observed earlier when Tcon were administered at day 0 (figure 2A), presumably due to a subsided inflammation that may influence the activation/proliferation of WT and Lag-3^−/−^ Tcon. The addition of WT Treg resulted in an increase in median survival of mice receiving Lag-3^−/−^ Tcon and Treg as compared to mice receiving Lag-3^−/−^ Tcon alone (45 days vs. 14.5 days, P = 0.008); however, WT Treg were considerably more effective at suppressing the proliferation of WT Tcon, and the addition of WT Treg to mice receiving WT Tcon improved the survival from a median survival of 26 days to >70 days (P = 0.0002). These results suggest that Lag-3 expression on Tcon might be part of the suppression mechanism employed by Treg.

### Lag-3^−/−^ Tcon Proliferate Faster than WT Tcon and have Enhanced Effector Function

**Figure 4:**
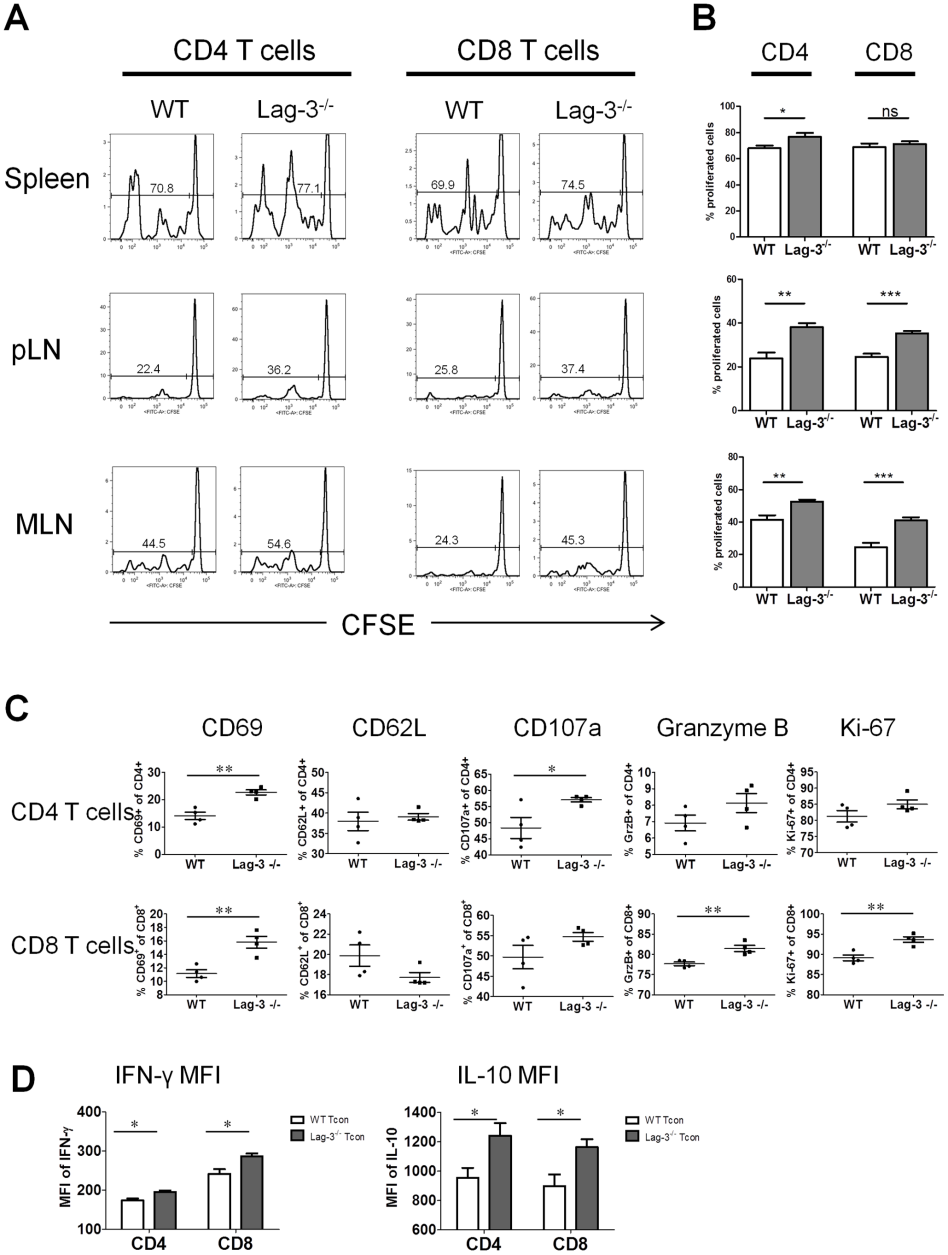
Lag-3^−/−^ Tcon show increased activation and proliferation. Lethally irradiated Balb/c recipients were transplanted with 5×10^6^ TCD-BM and 7.5×10^5^ CFSE-labeled WT or Lag-3^−/−^ Tcon. Donor T cells were re-isolated 3 days after transplant. (A) CFSE histograms of donor CD4 T cells (left panels) and CD8 T cells (right panels) re-isolated from spleen (top), pLN (middle), and MLN (bottom). Histograms are representative of 5 mice/group. Numbers represent percentage of proliferated cells. (B) Bar graphs indicating the percentage of proliferated donor CD4 T cells (left panels) and CD8 T cells (right panels) re-isolated from spleen (top), pLN (middle), and MLN (bottom). Bar depicts mean plus or minus SD, n = 5 mice/group. (C) and (D) Mice were transplanted with 5×10^6^ TCD-BM and 7.5×10^5^ WT or Lag-3^−/−^ Tcon. Donor T cells were re-isolated 4 days after transplant. To facilitate retention of intracellular cytokines *in vivo*, mice were injected i.p. with 250 µg brefeldin A 6 h before spleen and LN harvest. (C) Frequency of indicated activation markers gated on donor CD4 T cells (upper panels) and donor CD8 T cells (lower panels) re-isolated from spleen. (D) Mean fluorescence intensity (MFI) of IFN-γ and IL-10 gated on donor CD4 and CD8 T cells re-isolated from spleen. Bar depicts mean plus or minus SD, each data point represents two pooled mice, n = 4/group. Statistical significance indicated by *(* P<0.05, **P<0.01, ***P<0.001).

Since Lag-3 is involved in the regulation of T cell activation and proliferation, we hypothesized that the observed increase in GVHD pathogenicity could be due to increased proliferation and/or activation of Lag-3^−/−^ T cells. To test this hypothesis, 7.5×10^5^ CFSE labeled Lag-3^−/−^ or WT Tcon were transferred into lethally irradiated allogeneic Balb/c recipients together with 5×10^6^ TCD-BM. Donor T cells were re-isolated from pLN, MLN, and spleen on day 3 post-transplantation. CFSE staining of donor T cells revealed more proliferation of Lag-3^−/−^ T cells as compared to the WT T cells (Fig. 4A). A significantly higher percentage of proliferated Lag-3^−/−^ T cells were observed in pLN (38% vs 23%, P = 0.002) and MLN (52% vs 41%, P = 0.007), but not in the spleen (Fig. 4B). Both donor CD4 and CD8 T cells isolated from Lag-3^−/−^ Tcon recipients showed a more activated phenotype than those isolated from WT Tcon recipients. As indicated in Fig. 4C, there is a significant increase in the expression of CD69, CD107a, and cytolytic effector molecule granzyme B, and a decrease in CD62L expression, suggesting that Lag-3^−/−^ Tcon are more activated than WT Tcon. Although statistical significance was not achieved for some of the markers analyzed, we observed a tendency toward increased activation on both CD4 and CD8 T cells isolated from spleen and LN of Lag-3^−/−^ Tcon recipients (Fig. 4C and data not shown). To assess differences between WT and Lag-3^−/−^ Tcon in cytokine production *in vivo*, transplanted mice were injected i.p. with 250 µg brefeldin A 6 hours prior to harvesting spleen and LN for analysis. As shown in Fig. 4D, both Lag-3^−/−^ CD4 and CD8 T cells secrete significantly more IFN-g and IL-10 than WT CD4 and CD8 T cells indicating once again an increased effector function of the Lag-3^−/−^ Tcon (P<0.05).

**Figure 5:**
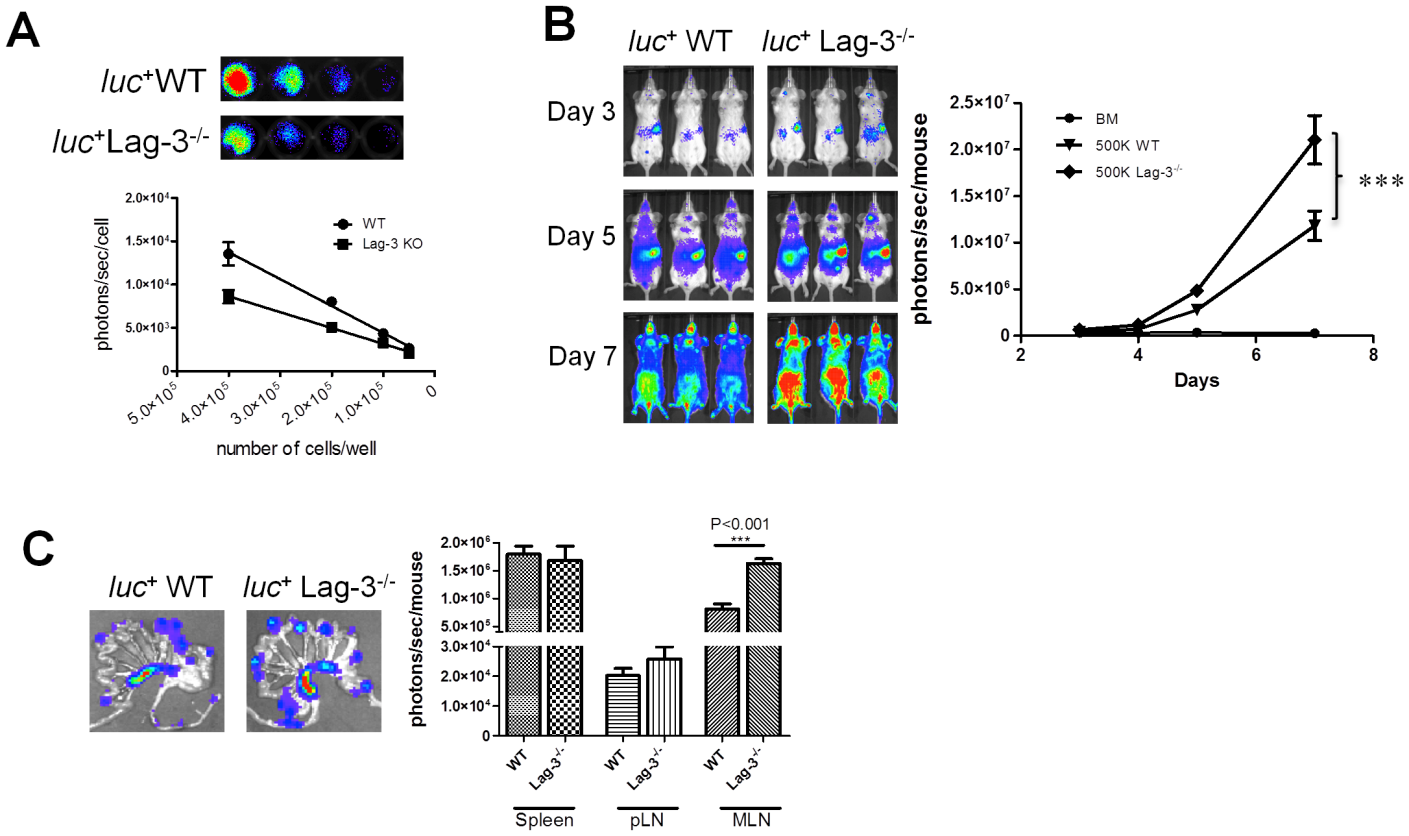
Lag-3^−/−^ Tcon proliferate faster than WT Tcon. (A) Quantitative analysis of photon emission *in vitro* by an equal number of *luc^+^* WT and *luc^+^* Lag-3^−/−^ Tcon. (B) Lethally irradiated Balb/c recipients were transplanted with 5×10^6^ TCD-BM and 5×10^5^ *luc^+^* WT or Lag-3^−/−^ Tcon. *In vivo* BLI images at different times after transplantation show more expansion of Lag-3^−/−^ Tcon. The graph on the right represents the quantitative analysis of the BLI signal. (C) *Ex vivo* images of the intestinal tract on day 4 displayed increased BLI signal in MLN of recipients receiving *luc^+^* Lag-3^−/−^ Tcon. The ex vivo images are representative of 5 mice/group. The bar graph on the right represents the quantitative analysis of the *ex vivo* BLI signal from spleen, pLN, and MLN. Bar depicts mean plus or minus SD, n = 5 mice/group. (*P<0.05, **P<0.01, ***P<0.001).

The difference in proliferation between Lag-3^−/−^ Tcon and WT Tcon was further confirmed *in vivo* using bioluminescence imaging (BLI). Donor T cells were isolated from either *luc^+^*C57BL/6 (WT *luc^+^*Tcon) or from Lag-3^−/−^ *luc^+^*C57BL/6 mice (Lag-3^−/−^ *luc^+^*Tcon) and their proliferation in allogeneic recipients was evaluated by whole body and *ex vivo* BLI. Due to a limited number of backcrossing of *luc^+^* WT C57BL/6 and Lag-3^−/−^C57BL/6 mice, there were differences in luciferase expression between Lag-3^−/−^ *luc* ^+^ and WT *luc* ^+^ mice that resulted in a 1.5 fold difference in BLI signal from an equal number of WT *luc^+^*Tcon and Lag-3^−/−^ *luc^+^*Tcon (Fig. 5A). In order to allow for a fair comparison between the two Tcon populations, the BLI results were adjusted to account for this difference. Whole body BLI indicated a significant increase in Tcon proliferation in mice receiving 5×10^5^ Lag-3^−/−^Tcon as compared to mice receiving an equal number of WT *luc^+^*Tcon (Fig. 5B, P<0.001). Similarly, *ex vivo* BLI imaging indicated differences in proliferation between Lag-3^−/−^Tcon and WT Tcon. A significant increase in BLI signal was detected in the pLN and MLN of mice receiving Lag-3^−/−^ *luc^+^*Tcon as compared to WT *luc^+^*Tcon (Fig. 5C). Taken together, these results suggest that increased proliferation and activation of Lag-3^−/−^ Tcon could account for the observed increase in GVHD pathogenicity.

### Lag-3^−/−^ Treg are Fully Functional

**Figure 6:**
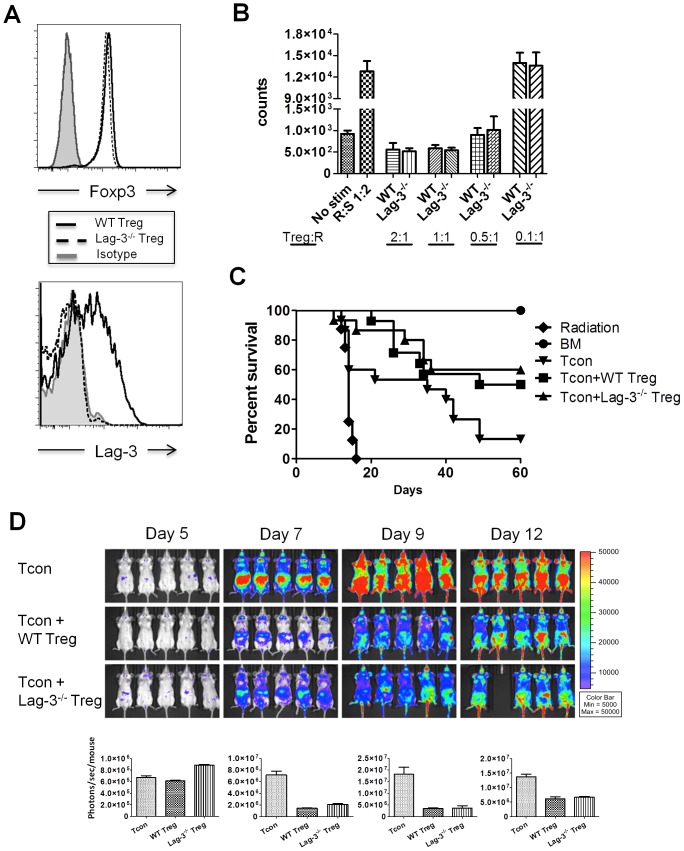
WT Treg and Lag-3^−/−^ Treg show similar protection against GVHD. (A) Foxp3 staining (upper panel) and Lag-3 staining (lower panel) of CD4^+^CD25^+^ T cells isolated from WT mice (black histogram) and Lag-3^−/−^ mice (dashed histogram). For Lag-3 staining, lower panel, regulatory T cells were activated for 2 days with anti-CD3/anti-CD28 beads. Cells were gated on CD4^+^Foxp3^+^. The shaded histogram represents the isotype staining control. The histograms are representative of 2 independent experiments. (B) ^3^H-thymidine incorporation of WT C57BL/6J responders to Balb/c stimulators in the presence of either WT Treg or Lag-3^−/−^ Treg at different Treg to responder ratios, R: responders, S: stimulators. (C–D) Lethally irradiated Balb/c recipients were co-transplanted with 5×10^6^ TCD-BM and 5×10^5^ WT or Lag-3^−/−^ Treg on day 0 followed by infusion of 1×10^6^ *luc^+^* WT Tcon on day 2. (C) Percent survival of mice after transplantation. Graph contains data pooled from 3 independent experiments (n = 15). P = 0.034 for WT Treg+Tcon vs. Tcon and P = 0.033 for Lag-3^−/−^ Treg+Tcon vs. Tcon. (D) *In vivo* BLI images of mice receiving WT Tcon alone, WT Tcon+WT Treg and WT Tcon+Lag-3^−/−^ Treg. Upper panels: BLI images taken at different time points after transplantation; lower panels: the corresponding quantitative analysis of the BLI signal. The BLI images are representative of 3 independent experiments.

Prior *in vitro* studies by Huang *et al*. suggested that Lag-3 is an essential molecule for the maximum suppressive function of Treg [19]. In mouse models of transplantation, we and others have shown that Treg suppress GVHD induced by donor allogeneic Tcon, making this a good model to explore the role of Lag-*3 in vivo* in Treg function [3], [7], [11], [12]. Therefore, by directly comparing Treg isolated from Lag-3^−/−^ donor mice to Treg isolated from WT donors, via *in vitro* and *in vivo* suppression assays, we sought to determine the role of Lag-3 in Treg function. Both WT and Lag-3^−/−^ Treg equally express Foxp3, and, as expected, WT Treg but not Lag-3^−/−^ Treg upregulate Lag-3 after *in vitro* stimulation with anti-CD3/anti-CD28 activation beads (Fig. 6A).

Next we compared Lag-3^−/−^ Treg to WT Treg in their ability to suppress the proliferation of alloreactive T cells in a mixed lymphocyte reaction. Treg from Lag-3^−/−^ mice efficiently suppress the proliferation of alloreactive T cells in a manner similar to WT Treg (Fig. 6B). Both Treg populations were able to extend the median survival of mice receiving Treg and Tcon as compared to mice receiving Tcon alone (60 days vs. 35 days, p = 0.03) (Fig. 6C). Likewise, when Lag-3^−/−^ Treg and WT Treg were tested *in vivo*, both were equally potent at suppressing Tcon proliferation as illustrated by whole body BLI analysis of mice receiving *luc^+^*Tcon (Fig. 6D). There was no difference in survival between groups receiving WT and Lag-3^−/−^ Treg, suggesting that Treg cell-intrinsic Lag-3 is not required for Treg function.

### Regulatory T Cells Acquire MHC Class II Complexes from Host APC

**Figure 7:**
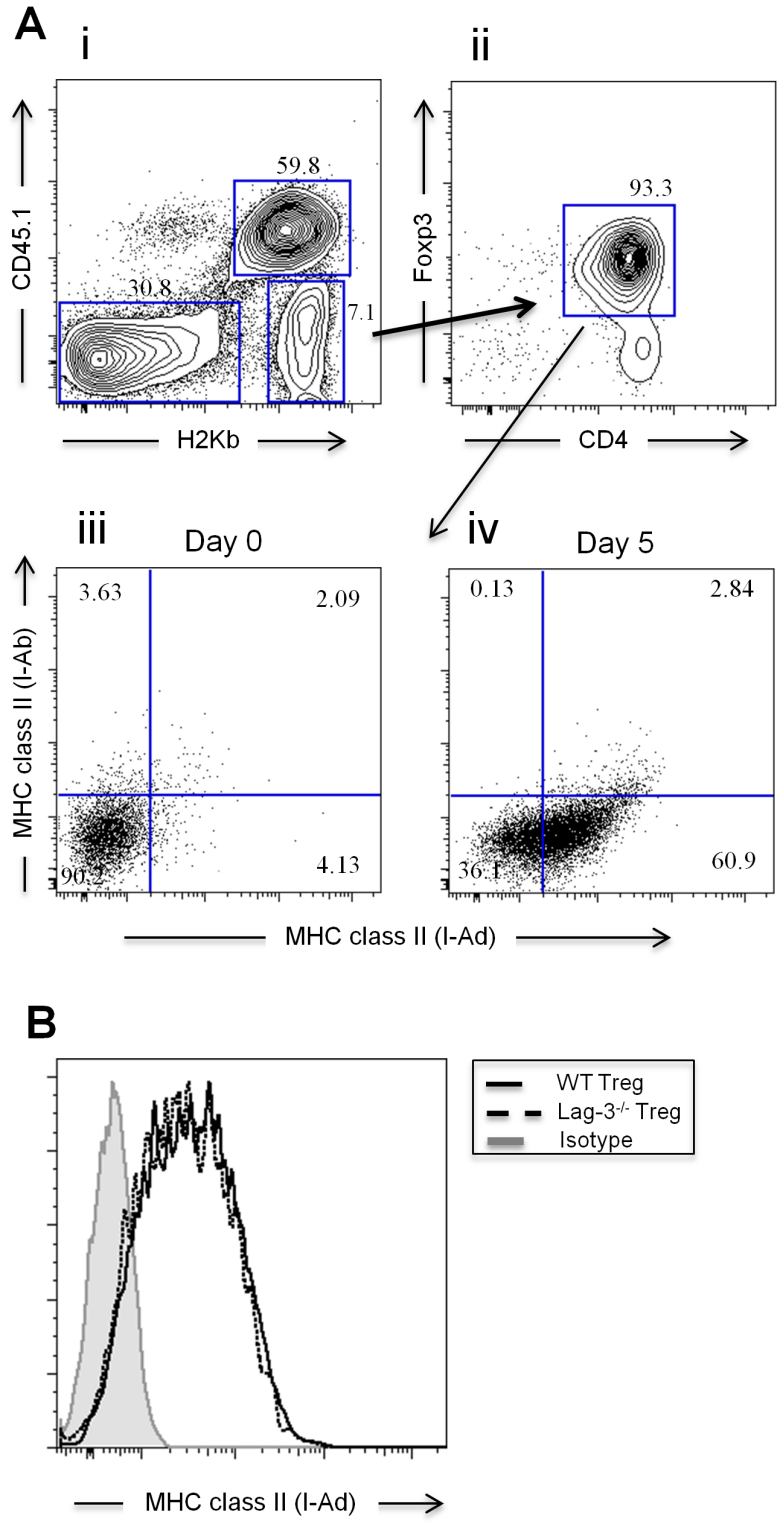
Regulatory T cells acquire MHC class II complexes from host APC. (A) Lethally irradiated Balb/c (H-2K^d^) recipients were co-transplanted with 5×10^6^ TCD-BM, 1×10^6^ Tcon from CD45.1^+^ C57BL/6 (H-2K^b^) mice and 1×10^6^ WT or Lag-3^−/−^ Treg. MHC class II expression on donor Treg was assessed 5 days after transplantation. Cells were gated on CD45.1 negative, H2Kb positive (panel i), then CD4 and Foxp3 positive events (panel ii). Panels iii and iv represent the MHC class II expression level on day 0 and day 5 respectively. (B) MHC class II staining on Treg of donor origin (WT or Lag-3^−/−^). Grey histogram represents cells stained with isotype control. Cells were gated on donor CD4 positive, Foxp3 positive events. The histograms are representative of 2 independent experiments each having 4 mice/group.

Our results so far indicate that Lag-3 does not play a significant role in Treg function since both WT Treg and Lag-3^−/−^ Treg equally suppress the proliferation of allogeneic Tcon. However, Treg were less able to suppress the proliferation of Lag-3^−/−^ Tcon (Fig. 3), suggesting that Lag-3 on T cells is necessary for optimal suppression by Treg. Since the known ligand for Lag-3 is MHC class II which mouse T cells do not express due to a lack in MHC class II transactivator (CIITA) transcripts [26] we hypothesized that Treg could acquire MHC class II from APC and subsequently use this molecule to suppress the proliferation of Lag-3 expressing Tcon. Prior studies have indicated that lymphoid cells are capable of acquiring important cell surface molecules upon interaction through a process termed trogocytocis [27]. To test this hypothesis, we transplanted lethally irradiated Balb/c mice with TCD-BM together with 1×10^6^ Tcon and 1×10^6^ WT or Lag-3^−/−^ Treg. The injected cells were re-isolated five days later based on congenic markers (Fig. 7A, panel i) and stained for MHC class II of donor origin (I-A^d^) or host origin (I-A^b^). As expected, we did not detect any MHC class II on Foxp3^+^CD4^+^ Treg on day 0 (Fig. 7A, panel iii); however, 5 days after transplant, an average of 65% of Foxp3^+^ Treg had acquired host MHC class II (Fig. 7A, panel iv). Both WT and Lag-3^−/−^ Treg equally acquired host MHC class II, suggesting once again that WT and Lag-3^−/−^ Treg are functionally similar (Fig. 7B).

## Discussion

Acute GVHD is characterized by activation of donor T cells when transferred in the conditioned host, followed by the proliferation and migration of T cells to target tissues including the skin, liver, and gastrointestinal tract. Since Lag-3 was up-regulated on activated T cells, we hypothesized that this cell surface transmembrane protein plays a role in GVHD pathophysiology. Donor T cells lacking Lag-3 resulted in accelerated GVHD progression with increased mortality in all of the T cell doses used to induce GVHD. Interestingly, even with very low dose of Tcon that typically induces only mild GVHD, if the T cells lack Lag-3, they induce aggressive GVHD resulting in 100% mortality. The increased aggressiveness of Lag-3^−/−^ Tcon is due to increased proliferation of these cells as illustrated by both CFSE staining and BLI analysis of *luc^+^*Lag-3^−/−^ Tcon and an increase in effector function as illustrated by higher levels of activation markers and cytokine production. Our data is consistent with studies from the Blazar group, which investigated the role of other inhibitory molecules such as CTLA-4, PD-1, and, most recently, Tim-3, in limiting the alloresponse in GVHD [28], [29]. Since the co-blockade of CTLA-4:B7 and PD-1:PD-1L interactions was additive in GVHD acceleration [28], suggesting the two pathways are not redundant, the engagement of the Lag-3 pathway may prove to be another mechanism of down-regulating proliferative T cell responses *in vivo.*

One could argue that the difference in proliferation between Lag-3^−/−^ and WT Tcon is not large enough to cause such a difference in GVHD manifestations. When Bettini *et al.* investigated the role of Lag-3 in the development of diabetes, while they did see an overall increase in Lag-3^−/−^ T cell proliferation, they observed a preferential expansion of islet Ag-specific clones compared to other infiltrating T cell clones [30]. Based on these data, we can speculate that, in our GVHD model, in the absence of Lag-3, the clones that expand the most are the most pathogenic clones, which are responsible for initiation and progression of GVHD and, therefore, more aggressive GVHD is observed.

We cannot rule out the possibility that other mechanisms in addition to increases in Tcon proliferation are responsible for the observed aggressiveness of Lag-3^−/−^ Tcon. Li *et al*. showed that the surface expression of Lag-3 is regulated by two transmembrane metalloproteases, ADAM10 and ADAM17, which cleave the extracellular domain of Lag-3 from the surface of activated T cells, releasing a soluble, momomeric, Lag-3 (sLag-3) [31]. The role of sLag-3 has not been fully elucidated. While Li *et al*. consider sLag-3 a “waste product” of Lag-3 cleavage with no immunological function due to its low affinity for MHC class II and rapid *in vivo* degradation [31], Triebel’s group showed that a sLag-3-Ig fusion protein up-regulated the expression of co-stimulatory molecules and increased IL-12 expression in dendritic cells, providing immune adjuvant activity [32]. In sharp contrast with this study, Buisson *et al*. demonstrated that sLag-3 impairs human monocyte differentiation into either macrophages or DCs. APC that differentiate from monocytes in the presence of GM-CSF, IL-4, and sLag-3 had weaker immunostimulatory capacities and therefore, a reduced capability to induce T cell proliferation [33]. We observed a gradual increase in Lag-3 expression on T cells immediately after activation with a maximum expression on day 4 post-transplantation, followed by a decrease in Lag-3 expression over the next several days. If the decrease in Lag-3 expression is due to the cleavage of Lag-3 by metalloproteases, then our data showing less proliferation of WT T cells and, consequently, less GVHD symptoms, would support the hypothesis that sLag-3 reduces DC differentiation from monocyte precursors. Further studies are needed to address this question.

Another potential mechanism that could explain the aggressiveness of Lag-3^−/−^ Tcon comes from studies by Okamura *et al*., which identified a novel regulatory T cell population, CD4^+^CD25^−^Lag-3^+^ T cells, that suppress T cell proliferation in an IL-10-dependent and FoxP3-independent manner [34]. The CD4^+^CD25^−^Lag-3^+^ T cells do not develop through thymic selection, but rather the environmental microbiota in the periphery was responsible for the development of CD4^+^CD25^−^Lag-3^+^ T cells [34]. In light of the findings in this study, and given the fact that GVHD involves gastrointestinal tract injury, with concomitant release of gut microbiota, we can speculate that a fraction of the Lag-3 expressing Tcon would convert into CD4^+^CD25^−^Lag-3^+^ regulatory T cells that would suppress to some extent donor T cell proliferation which could lead to an alleviation of GVHD symptoms. One could speculate that donor T cells lacking Lag-3 are incapable of conversion into CD4^+^CD25^−^Lag-3^+^ regulatory cells, therefore, resulting in more aggressive GVHD. The hypothesis of peripheral conversion into CD4^+^CD25^−^Lag-3^+^ IL-10-secreting regulatory T cells can also explain why a combination WT-CD4 and Lag-3^−/−^CD8 T cells did not cause an increase in GVHD pathogenicity while the combination of Lag-3^−/−^CD4 and WT-CD8 T cells did (Fig. 2G).

Studies by Huang *et al*. indicate that Lag-3 is up-regulated on induced Treg (iTreg) and is required for the iTreg function *in vitro* and *in vivo* [*19*]. Lag-3 was also up-regulated on natural Treg and seemed to affect the suppressive ability of Treg *in vitro* in a mixed lymphocytes assay with irradiated C57BL/6 APC as stimulators, CD4^+^CD25^−^OT-IILag-3^−/−^ T cells as responders and increasing concentration of OVA peptide [19]. Interestingly, there was no difference between Lag-3^+^ and Lag-3^−/−^ Treg in suppression of T cell proliferation at low doses of OVA peptide, but only at high doses [19]. Anti-Lag-3 antibody reduced the suppressive function of iTregs; however, the effect of anti-Lag-3 antibody on Treg function was more pronounced at a lower Treg to responder ratio (1∶250 to 1∶10 Treg:Responder ratio). When more iTregs were used (1∶2 ratio of Treg to responders) the effect of anti-Lag-3 antibody was abolished. Similar to these studies, we also observed up-regulation of Lag-3 expression on natural CD4^+^CD25^high^Foxp3^+^ Treg. However we did not detect any differences in suppression of proliferation of donor T cells by Lag-3^−/−^ Treg either *in vitro* or *in vivo*. We believe the observed differences between our results and Huang’s published results lay in the experimental setup. We are using a much higher Treg to responder ratio in our suppression assay that is placing our results to the end of Huang’s curve where there was no difference in suppression in the presence or absence of functional Lag-3 on the surface of Treg. Since Tregs utilize different molecules to mediate suppression, it is possible that the observed role that Lag-3 plays at lower Treg numbers is masked by a more dominant mechanism independent of Lag-3 at higher Treg numbers.

Several different mechanisms of suppression for Treg have been proposed, including the secretion of immunosuppressive cytokines (IL-10, TGF-β, IL-35) [35], [36], [37], [38], granzymes, and perforin [39], [40], aggregation around APC followed by functional modification of APC and suppression through direct contact with effector cells [41], [42]. A working model for Treg-mediated suppression that seems widely accepted involves three steps: (1) upon antigen stimulation, Treg aggregate around antigen presenting DCs preventing naïve T cell activation; (2) while in contact with DC, Treg down-regulate co-stimulatory molecules CD80/CD86, preventing activation of other T cells by DC; and (3) after leaving DC, Treg will further differentiate to secrete granzyme/perforin or immunosuppressive cytokines. A recent study by Zhou *et al*. indicated that Treg are able to acquire peptide-MHC class II complexes from APC through a process termed trogocytosis [43]. Treg with acquired MHC-II have enhanced suppression potency and can suppress the proliferation of naïve T cells in an APC-free experimental setting [43]. MHC class II is the known ligand for the inhibitory receptor Lag-3. We show here that donor Treg, both WT and Lag-3^−/−^, can acquire host MHC class II and therefore, propose that the engagement of Lag-3 on Tcon by the MHC class II on Treg may regulate the expansion of activated T cells. This interaction could be an additional, novel mechanism of suppression employed by Treg. Thus, these findings could explain why Treg are less effective at suppressing the proliferation of Lag-3^−/−^Tcon, resulting in increased GVHD symptoms and decreased survival as compared with the suppression of WT Tcon. Additionally, Treg that lack Lag-3 on their surface have similar suppression potency as WT Treg suggesting that the existence of Lag-3 on Tcon is important for the suppression activity and that Lag-3^−/−^ Treg are fully functional.

In conclusion, our results demonstrate that Lag-3 is an important regulatory molecule involved in alloreactive T cell proliferation after BMT. Furthermore, based on our results with Treg, we propose a more detailed suppression model of how Treg exert their suppressive function. The model that we propose could be amended to the following: (1) Treg aggregate on DC; (2) Treg down-regulate costimulatory molecules on DC; (3) Treg acquire MHC-II-peptide complexes from DC through trogocytosis; and (4) MHC-II on Treg interact with Lag-3 on activated T cells to exert their suppressive function.
